# IL-33 genetics and epigenetics in immune-related diseases

**DOI:** 10.1186/s12948-021-00157-6

**Published:** 2021-09-26

**Authors:** Eleonora Di Salvo, Marco Casciaro, Sebastiano Gangemi

**Affiliations:** 1 Department of Veterinary Sciences, University of Messina, Messina, Italy; 2 School and Operative Unit of Allergy and Clinical Immunology, Department of Clinical and Experimental Medicine, Policlinico “G. Martino“, University of Messina, Messina, Italy

**Keywords:** IL-33, genetics, epigenetics, inflammation, immune system

## Abstract

Interleukin-33 (IL-33) is a 30KDa protein, which belongs to the Interleukin-1 cytokine family. It is a crucial regulator of innate and adaptive immune responses. This interleukin is additionally involved in the inflammatory reaction versus helminthic infections. Interleukin 33 acts on group 2 innate lymphoid cells and mast cells macrophages, dendritic cells and CD4 + Th2 cells eliciting a type 2 immune response. Moreover, the cytokine can activate the ST2 of Tregs, demonstrating its ability to downregulate inflammation. IL-33 has also an intracellular function by regulating transcription. The active IL-33 doesn’t have a signal peptide, so it’s not released across a normal secretory pathway; the interleukin is released when the cells are damages and acts like an “alarmin”. Its influence on immune activation could be slightly adjusted via fine epigenetic interactions involving cascade pathways and immune genes. Due to the diverse data emerged from different experimental research, we decided span literature to clarify, as much as possible, how IL-33 is influenced by and influence gene expression. The authors reported how its balance is influenced, according to the tissue considered. Fundamental for immune-related diseases, IL-33 has a key role in controlling inflammation. The understanding of the cytokine switch will be fundamental in a near future in order to block or activate some immune pathways. In fact, we could control interleukins effects not only by monoclonal antibodies but also by using siRNA or miRNAs for silencing or expressing key genes.

To the Editor,

interleukin-33 (IL-33) is a 30KDa protein, which belongs to the Interleukin-1 (IL-1) cytokine family. It is a crucial regulator of innate and adaptive immune responses. This interleukin is additionally involved in the inflammatory reaction versus helminthic infections [1, 2]. IL-33 exerts its role as an extracellular signal binding to a heterodimeric receptor complex combined by ST2 (also known as IL1RL1) and by IL-1 receptor accessory protein (IL-1RAcP) [3, 4]. Interleukin 33 acts on group 2 innate lymphoid cells (ILC2) and mast cells [5], macrophages, dendritic cells and CD4 + Th2 cells eliciting a type 2 immune response. Moreover, the cytokine is able to activate the ST2 of Tregs, demonstrating its ability to downregulate inflammation [2]. IL-33 could be found in different tissues such as in epithelial, lung, epidermal, gastrointestinal, reproductive ones [6]. IL-33 is additionally highly represented in diverse other cells [7]. IL-33 has also an intracellular function by regulating transcription. It is protein with two domains: the C-terminal (aa 112–270 in humans) and the N-terminal domain (aa 1-111 in humans). The first one contains IL-1 family member homology and mediates the extracellular, ST2-dependent effects. The second one matches IL-33 to the nucleus, made by a chromatin-binding motif, and demonstrates transcriptional repressor action in an artificial tethered gene reporter assay [8]. Several authors tried to delineate IL-33 target genes; some of these targets were IL-6 and RELA (NF-kB p65) [9, 10]. The IL-33 chromatin-binding motif facilitates the binding to histone dimers and modifies the chromatic structure. This process is involved in the transcription of genes, interfering with gene repression [8]. The active IL-33 doesn’t have a signal peptide, so it’s not released across a normal secretory pathway; the interleukin is released when the cells are damages and acts like an “alarmin” [11, 12]. It has multiple functions. In fact, in the nucleus it acts on tissue modelling and repair; on the other hand, if IL-33 is secreted extracellularly it has pro-inflammatory effects. In these cases, immune activation could be slightly adjusted via fine epigenetic interactions involving cascade pathways and immune genes [13]. Chromatin epigenetic modifications capable of influencing gene expression [14] comprise methylation of DNA, post- translational modifications of histone tails (i.e. acetylation and methylation). The results are an augmented or a reduced access of transcriptional factors to gene promoters and enhancers capable of modulating inflammation [15]. Due to the diverse data emerged from experimental research, we decided span literature to clarify, as much as possible, how IL-33 is influenced by and influence gene expression.

We collected some of the most relevant articles in Table 1.

Several authors took in consideration the effects of IL-33 on genetic expression by acting either directly or indirectly. We retrieved some of these pathways. Apigenin and luteolin were able to suppress the production of IL-33 by inhibiting its gene and protein expression in the microglia cells. They acted mainly on MAPKs, NF-κB, and STAT3 signalling pathways in LPS-activated microglial cells [16]. Also, the chromatin remodeling protein, BRG1, possesses the ability to regulate the transcription of IL-33 in endothelial cells. BRG1 lack improved renal inflammation by diminishing IL-33 production [17]. Histone deacetylase (HDAC3) is an enzyme that act a role in the epigenetic balance. HDAC3 acts by transcriptional repressor capable of influencing IL-33 expression [18]. Some authors evaluated genes associated to IL-33 expression (DND1, PET100, GPR160, LPAR6, and SERTAD3) and HDAC3/HDAC1 in patients affected by multiple sclerosis (MS). IL-33 was highly correlated to multiple protein-coding genes in the relapse-remission cohort of patients. However, these genes, but not IL-33, were involved in DNA repair or mitochondrial function and mRNA splicing pathways [19]. Dusp (Dual-specificity phosphatase), is a form of phosphatase that can act upon tyrosine or serine/threonine residues. Dusp5 mRNA was highest in eosinophils and NK cells and was upregulated by IL-33. IL-33-activated Dusp5/ eosinophils had improved cellular ERK1/2 activation and BCL-XL expression. The consequence was higher eosinophil survival [20]. On the other hand, IL-33 can induce IL-8 gene and protein expression through the activation of JNK/c-Jun/AP-1 pathway. IL-8 production via JNK-c-Jun-AP-1 pathway, cause inflammatory syndromes [21]. The regulation of genetic expression passes also through silencing RNAs so they were reported being able to silence Rip2 which in turn blocked mRNA expression of ICAM-1, VCAM-1, E-selectin, RANTES, IL-17, IL-33, thymic stromal lymphopoietin, inducible NO synthase, and MUC5ac in lungs [22]. Also, NOD-like receptor protein 3 (NLRP3) gene silencing, influenced IL-33 as it reduced the production of IL-1β, IL-18, and IL-33 [23]. Recently, it was also demonstrated that IL-33 in Alzheimer’s Disease ameliorated Aβ pathology by reprogramming microglial epigenetic and transcriptomic profiles. IL-33 remodelled chromatin accessibility and PU0.1 transcription factor binding. The PU0.1-dependent transcriptome reprogramming was fundamental for the IL-33-induced Aβ clearance [24]. Epigenetic importance on IL-33 production was also demonstrated with sublingual immunotherapy. In fact, recombinant Che a 2 (rChe a 2), a major allergen of Chenopodium album, exposure diminished both mRNA and protein levels of IL-33, by induction of distinct histone modifications at specific loci [25]. Other results demonstrated that IL-33 could exerts anti-apoptotic influence by inhibiting the PKCβ/ c-Jun N-terminal kinase (JNK) pathway [26].

**Table 1:** List of the main articles evaluating how IL-33 is influenced by and influence gene expression

Reference	Disease	Tissue	Genetic pathways	Effects
[16]	-	CNS / microglia	Apigenin and luteolin inhibit Iba-1	They act on IL-31 and NF-κB by reducing IL-33 production
[17]	Ischemia-reperfusion, renal injury	Kidneys	The chromatin remodeling protein BRG1	It regulates the transcription of IL-33
[18]	MS	PBMC cells	HDAC3	It acts by transcriptional repressor of IL-33
[19]	MS	CNS	DND1, PET100, GPR160, LPAR6, and SERTAD3 correlate with IL-33	These genes, but not IL-33, are involved in DNA repair or mitochondrial function and mRNA splicing pathways.
[20]	Infection of *Mesocestoides corti* and *Nippostrongylus brasiliensis*	Bone marrow (eosinophil)	Dusp5	IL-33-activated Dusp5/ eosinophils had improved cellular ERK1/2 activation and BCL-XL expression resulting in higher eosinophil survival
[21]	Atherosclerotic and inflammatory diseases	HUVECs cells	IL-8 gene	IL-33 induces IL-8 expression through the activation of JNK/c-Jun/AP-1 pathway causing inflammatory syndromes
[22]	Allergic asthma	Lung	siRNA blockade of Rip2	Therefore, it blocks the expression of IL-33 ameliorating inflammation
[23]	Acute lung injury	Lung	NLRP3 silencing	Reduce IL-33 expression
[24]	Alzheimer’s disease	Brain/microglia	IL-33-provoked remodeling of chromatin accessibility and PU0.1 transcription factor binding	Modify microglial epigenetic and transcriptomic profiles resulting in Alzheimer amelioration
[25]	Allergic diseases	Lung	Trimethylated lysine 27 of histone H3 at promoter regions of IL-33	Down-regulation of IL-33
[26]	Cardiovascular diseases	Heart	PKCβ/JNK	IL-33 inhibits apoptosis

*CNS* central nervous system, *HDAC* histone deacetylase, *MS* mutiple sclerosis, *PBMC* peripheral blood mononuclear cells, *NF-kB* nuclear factor kappa-light-chain-enhancer of activated B cells, *NLRP3* NOD-like receptor protein 3; *AI* airway inflammation; IL: interleukin, *TSLP* Thymic stromal lymphopoietin, *JUN* c-Jun N-terminal kinase, *Dusp* Dual-specificity phosphatase

Epigenetic regulation of immune cell behaviour is becoming increasingly accepted as a likely mechanism by which immune cell subsets mediate responses to widely differing stimuli [15]. IL-33 is arousing lot of interest due to its action on the immune system both as regulator and as pro-inflammatory cytokine. The authors cited above reported how its balance could is influenced in different ways, according to the tissue considered. Fundamental for immune-related diseases, IL33 has a key role in controlling inflammation. On one side, Apigenin, Luteolin and immunotherapies demonstrated they efficacy in reducing the interleukin expression by acting on MAPKs, NF-κB, and STAT3 [16, 25]. On the other side, most of the authors reported that several genes (BRG1, HDAC3, DND1, PET100, GPR160, LPAR6, SERTAD3, Rip2, NLRP3) are linked to IL-33 levels, although the exact correlation have to be clarified [17–19, 22, 23]. Data demonstrated that the alarmin could also act on several genes exerting diverse cascades influencing inflammation as well apoptosis. In fact, IL-33 was demonstrated binding transcription factors, interfering with chromatine accessibility [24]. Some of the interleukin epigenetics effects were also favoring cells survival and immortalizing some others via PKCβ/JNK, ERK1-2 and BCL-XL [20, 26]. Some of the genes influencing or influenced by IL-33 were retrieved in Fig. 1.

IL-33 appears to be central in balancing immune response. It acts as a danger signal and as an immune-mediator. It effects could be direct on gene expression or indirect by influencing other cytokines and cells recruitment. Figure 1 aims to summarize some of the pleiotropic genetic and epigenetics of IL-33. A better understanding of this cytokine switch will be fundamental in a near future to block or activate some immune pathways. Next achievement should monoclonal antibodies against IL-33 to block the effects of the alarmin once released. One further step should also include siRNA or miRNAs for an early silencing of key genes involved in the interleukin production.

**Fig. 1:**
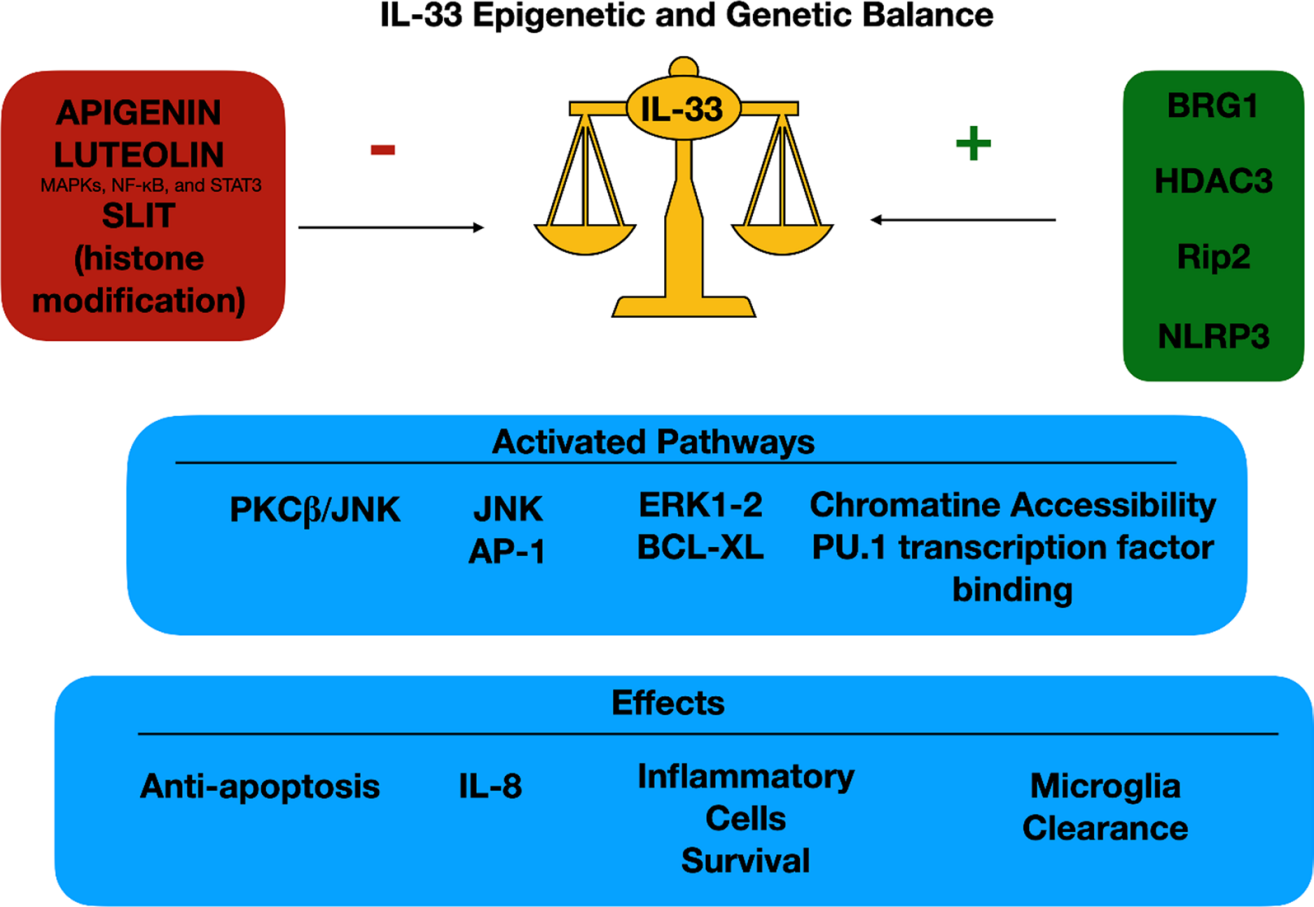
IL-33 epigenetic and genetic balance

## Data Availability

Not applicable.
